# An old way of forgetting

**DOI:** 10.1192/j.eurpsy.2022.1681

**Published:** 2022-09-01

**Authors:** J.P. Rebeca Coelho

**Affiliations:** Centre Neuchâtelois de Psychiatrie, Cnpa1, Neuchâtel, Switzerland

**Keywords:** Syphilis, Dementia

## Abstract

**Introduction:**

During the last decades, the incidence of syphilis is on the rising, particularly in the United States of America and Europe. Neurosyphilis is a disease that has a vast differential diagnostic. With that in mind, clinicians have some difficulties to identify it rapidly. A case of a 57-year-old man is presented, with a brutal change in his behavior, associated with a dementia-like syndrome. He is diagnosed with neurosyphilis.

**Objectives:**

The main goal is to present his clinical psychiatric symptoms and diagnosis procedure, the treatment that he received and his clinical outcome in the psychogeriatric department.

**Methods:**

The treatment was based in an integrated framework of pharmacology and psychotherapy.

**Results:**

The patient was able to slowly recover and to get back home, we a solid structure to make the follow up.

**Conclusions:**

This clinical vignette represents a growing number of adult patients that present themselves for the first time with dementia-like symptoms. It is important to remember, that many diseases are capable of mimicking dementia and their exclusion before admitting a diagnosis of dementia is mandatory.

**Disclosure:**

No significant relationships.

